# Exploration of a novel virtual environment improves memory consolidation in ADHD

**DOI:** 10.1038/s41598-020-78222-4

**Published:** 2020-12-08

**Authors:** Valentin Baumann, Thomas Birnbaum, Carolin Breitling-Ziegler, Jana Tegelbeckers, Johannes Dambacher, Elke Edelmann, Jorge R. Bergado-Acosta, Hans-Henning Flechtner, Kerstin Krauel

**Affiliations:** 1 Department of Child and Adolescent Psychiatry and Psychotherapy, University of Magdeburg, Leipziger Straße 44, 39120 Magdeburg, Germany; 2 Department of Neurology, Feinberg School of Medicine, Northwestern University, Chicago, IL USA; 3 Faculty of Computer Science, University of Magdeburg, Magdeburg, Germany; 4 Department of Physiology, University of Kiel, Kiel, Germany; 5 Department of Pharmacology and Toxicology, University of Magdeburg, Magdeburg, Germany; 6 Center for Behavioral Brain Sciences, Magdeburg, Germany

**Keywords:** Human behaviour, ADHD, Consolidation, Hippocampus, Long-term memory, Translational research

## Abstract

Experimental evidence in rodents and humans suggests that long-term memory consolidation can be enhanced by the exploration of a novel environment presented during a vulnerable early phase of consolidation. This memory enhancing effect (behavioral tagging) is caused by dopaminergic and noradrenergic neuromodulation of hippocampal plasticity processes. In translation from animal to human research, we investigated whether behavioral tagging with novelty can be used to tackle memory problems observed in children and adolescents with attention-deficit/hyperactivity disorder (ADHD). 34 patients with ADHD and 34 typically developing participants (age 9–15 years) explored either a previously familiarized or a novel virtual environment 45 min after they had learned a list of 20 words. Participants took a free recall test both immediately after learning the word list and after 24 h. Patients who explored a familiar environment showed significantly impaired memory consolidation compared to typically developing peers. Exploration of a novel environment led to significantly better memory consolidation in children and adolescents with ADHD. However, we did not observe a beneficial effect of novel environment exploration in typically developing participants. Our data rather suggested that increased exploration of a novel environment as well as higher feelings of virtual immersion compromised memory performance in typically developing children and adolescents, which was not the case for patients with ADHD. We propose that behavioral tagging with novel virtual environments is a promising candidate to overcome ADHD related memory problems. Moreover, the discrepancy between children and adolescents with and without ADHD suggests that behavioral tagging might only be able to improve memory consolidation for weakly encoded information.

## Introduction

Attention-deficit/hyperactivity disorder (ADHD) is one of the most common psychiatric disorder in childhood and adolescence^1^. Typically, patients suffer from persisting attentional problems, impulsive behaviors and hyperactivity^2^. Reduced attentional capacities in ADHD are closely linked to impairments in working memory, the ability to temporarily hold and manipulate information^3^. However, ADHD is also associated with compromised formation and retrieval of episodic memory^4–12^, which describes long-term memory for every day events and their contextual details^13^. Individuals with ADHD face particular difficulties when they cannot rely on external cues and have to retrieve information in free recall^4,5^. These memory deficits are mainly observed in immediate retrieval, indicating that memory encoding is impaired^6–9^. However, deficits are also evident during memory consolidation as shown in an additional decrease in memory performance in delayed retrieval both after short intervals of 20–30 min^4,5,10,11^ and longer intervals of 12 h^12^.

One way to improve specifically memory consolidation is behavioral tagging^14–16^. Behavioral tagging exploits the phenomenon that there is a critical period after memory encoding when memory traces are still malleable and can be influenced by other experiences^17,18^. For example, in animal experiments the exploration of a novel, but not a familiar environment reliably enhances memory consolidation in an unrelated learning task, as long as the novel experience is presented within a critical period after initial learning^19–22^. On a cellular level, behavioral tagging is explained through a synaptic tagging and capture (STC) mechanism^23–25^.

According to STC theory, memory consolidation depends on a tagging process that marks the synapses forming a memory trace as well as the synthesis and subsequent capture of plasticity related proteins (PRPs) at the tagged synapses. Weak learning events set a synaptic tag but generate an insufficient amount of PRPs, which results in intact short-term memory (STM), but subsequent forgetting. In contrast, strong learning events provide sufficient PRPs to support further plasticity processes that ultimately create a stable long-term memory (LTM) trace. However, tags set by a weak learning event may “hijack” PRPs generated by a strong event. Since synaptic tags persist for up to 1–2 h^26–28^, the presentation of a salient, strong event is able to improve the consolidation of a previously encoded weak memory trace.

Recent evidence indicates that experiencing novelty also influences memory in humans^29–32^. In an experiment with typically developing elementary school children, Ballarini et al.^29^ showed that exposure to a novel school lesson was able to improve 24 h delayed cued recall of a previously presented story. Importantly, the novel school lesson and the story were unrelated in content and the novel lesson improved memory of the story even if presented 1 h before or after memory encoding. Recently, this effect was replicated in a sample of high school students, where 24 h LTM of a spatial memory task was improved by a novel school lesson presented 1 h before or after encoding^30^. Together with animal experiments, these studies indicate that the critical period during which behavioral tagging may occur around 30 to 60 min before or after learning^14^.

So far, experiments with children and adolescents used very elaborate novel experiences, for example combining the novelty of an unfamiliar topic with a new teacher and with the spatial novelty of an unknown room^29,30^. A recent experiment indicated that a similar effect could be achieved using exploration of a novel virtual environment^31^. Since the exploration of a novel environment is the most common way to induce behavioral tagging in animal experiments, virtual environments allow a close translation to human research^33–35^. In humans, some studies suggest that active rather than passive exploration of a novel experience might be needed to improve memory^36,37^. However, self-guided exploration might cause the actual experience of a novel virtual environment to vary greatly between individuals, for example due to how participants navigate or how deeply they immerse themselves in the virtual world^38–40^. In previous studies, both the level of immersion and the magnitude of exploration were shown to be positively associated with performance in unrelated memory tasks^31,35^.

In the current experiment, we aimed to exploit behavioral tagging to enhance long-term memory consolidation (24 h) in children and adolescents with ADHD. Specifically, we intended to improve intentional learning of a word list through the active exploration of a novel virtual environment. Intentional word list learning is an established, ecologically valid memory paradigm that mimics common learning situations encountered in school, for example when tasks demand encoding of history facts or the acquisition of foreign vocabulary. To ensure that our manipulation truly affected memory consolidation and to exclude mere attentional or arousal effects we presented the novel environment 45 min after memory encoding. Regarding the measurement of memory, we chose free recall as our primary outcome variable, as free recall is the memory measure were patients with ADHD struggle the most^4,5^. We expected both children and adolescents with ADHD as well as a control group of typically developing (TD) children and adolescents to show better memory consolidation if a novel environment was explored (*ADHD-novel* and *TD-novel*) compared to when a familiar environment was explored (*ADHD-familiar* and *TD-familiar*). Since the effect of a novel virtual environment might be modulated by how it is experienced individually, we moreover assessed movement data during exploration, feeling of immersion and the individual attitude towards novelty exploration.

## Methods

### Participants

In total, 72 children and adolescents aged between 9 and 15 years participated in the experiment. Patients were recruited via the department of child and adolescent psychiatry and psychotherapy, licensed pediatricians or child psychiatrists as well as advertisements within the local community. Typically developing children and adolescents were also addressed via local advertising (e.g. newspaper, postcards, leaflets). All participants and their parents were interviewed by trained psychologists using the German adaption^41^ of the Revised Schedule for Affective Disorders and Schizophrenia for School-Age Children: Present and Lifetime Version (K-SADS-PL)^42^. Patients meeting present or lifetime criteria for any psychiatric disorder other than oppositional defiant disorder (ODD), conduct disorder (CD) or enuresis were excluded from the sample. Typically developing controls were excluded if there was any evidence of previous or current psychiatric disorders. Any past or present neurological disorder or substance abuse served as exclusion criteria for both groups, as well as an IQ below 80. The Child Behavior Checklist (CBCL)^43^, the Youth Self Report (YSR)^44^ and the Diagnosis Checklist for Disruptive Behavior Disorder from the German Diagnostic System for Mental Disorders in Children and Adolescents (DISYPS-III)^45^ were used as additional clinical measures. To collect supportive diagnostic information, standardized measures of intelligence (Culture Fair Intelligence Test, CFT 20-R)^46^, attention (selective attention: d2-R^47^; alertness: Testbatterie zur Aufmerksamkeitsprüfung^48^), behavioral control (Go/No-Go: Testbatterie zur Aufmerksamkeitsprüfung^48^) and episodic memory (Verbal Learning and Memory Test, VLMT)^49^ were obtained. Handedness was determined with the Edinburgh Handedness Inventory^50^. The integration of the available diagnostic information was conducted under the supervision of a licensed child and adolescent psychotherapist (KK). 34 participants met the diagnostic criteria for ADHD as required by the Diagnostic and Statistical Manual of Mental Disorders (DSM-5)^51^ (24 combined presentation, 9 predominantly inattentive presentation, one predominantly hyperactive-impulsive presentation). Seven patients were additionally diagnosed with ODD. Children receiving stimulants were required to refrain from medication at least 24 h before the experiment and for the entire duration of the experiment (day 1 to 3). As the half-life of methylphenidate is about 2 to 3 h^52^, withholding methylphenidate intake for 24 h was considered sufficient to reach an off-medication baseline at the start of the experiment. Four participants were excluded due to technical issues or incompliance during the experiment. After exclusion, 68 participants remained in the sample (for sample characteristics, see Table 1). All received 20€ in vouchers for their participation.

**Table 1:** Group characteristics.

	TD-familiar	TD-novel	ADHD-familiar	ADHD-novel	Contrasts
n	17	17	17	17	
Age (years)	12.4 (1.9)	11.8 (1.6)	11.9 (2.1)	12.4 (1.8)	n.s.
Sex (male:female)	13:4	13:4	15:2	14:3	
CFT 20-R (IQ)	108.8 (13.3)	114 (12.8)	98.9 (10.6)	98.8 (16.0)	TD_nov > ADHD_fam, ADHD_nov
**VLMT (T-values)**					
Immediate recall	55.5 (10.6)	50.0 (9.9)	41.4 (6.6)	47.1 (9.2)	TD_fam > ADHD_fam
LTM recall (30 min)	55.4 (9.5)	54.2 (6.9)	48.5 (8.1)	46.3 (9.3)	TD_fam > ADHD_nov
LTM recognition (30 min)	55.1 (12.7)	53.5 (12.1)	47.9 (9.3)	50.9 (11.6)	n.s.
**ADHD presentation**					
Hyperactive	–	–	1	0	
Inattentive	–	–	4	5	
Combined	–	–	12	12	
Comorbid ODD diagnosis	–	–	4	3	
DISYPS-III	0.36 (0.29)	0.25 (0.48)	1.58 (0.52)	1.56 (0.24)	TD_fam, TD_nov < ADHD_fam, ADHD_nov
Play videogames (never/1–2 × month/1–2 × week/daily)	0/1/5/11	1/2/8/6	1/3/8/5	1/2/2/12	n.s.
Play Minecraft (never/1–2 × month/1–2 × week/daily)	7/1/6/3	3/6/6/2	2/6/8/1	4/4/6/3	n.s.
know Minecraft (yes:no)	17:0	17:0	17:0	16:1	

Data reflects sample after participant exclusion. If not specified otherwise, values represent either mean and standard deviation (in brackets) or counts. The parameters *play videogames* and *play Minecraft* represent the previous experience with videogames in general and Minecraft in particular. The parameter *know Minecraft* refers to how many children had at least seen someone else play the videogame before, and were thus familiar with its general appearance. To check for differences between groups, we computed a one-way ANOVA with the four groups (*TD-familiar, TD-novel, ADHD-familiar, ADHD-novel*) as the single factor. Significant contrasts show the results of Bonferroni or, in case of unequal variances, Games-Howell corrected post hoc tests. In case of ordinal data, we applied the Kruskal–Wallis test instead of an ANOVA. All tests were conducted with an alpha level of α = .05. Abbreviations: TD = Typically Developing, CFT = Culture Fair Intelligence Test, VLMT = Verbal Learning and Memory Test, ODD = oppositional defiant disorder, DISYPS = Diagnostic System for Mental Disorders in Children and Adolescents (Diagnosis Checklist for Disruptive Behavior Disorder).

Children and their parents received oral and written information about the contents of the study beforehand. Both were informed that their participation was voluntary and that they could withdraw from the experiment at all times. All children and adolescents gave written informed assent and parents gave written informed consent to the participation of their children. The experiment was approved by the local ethics committee of the University of Magdeburg, Faculty of Medicine, and followed the ethical standards of the Declaration of Helsinki.

### Procedure

**Figure 1:**
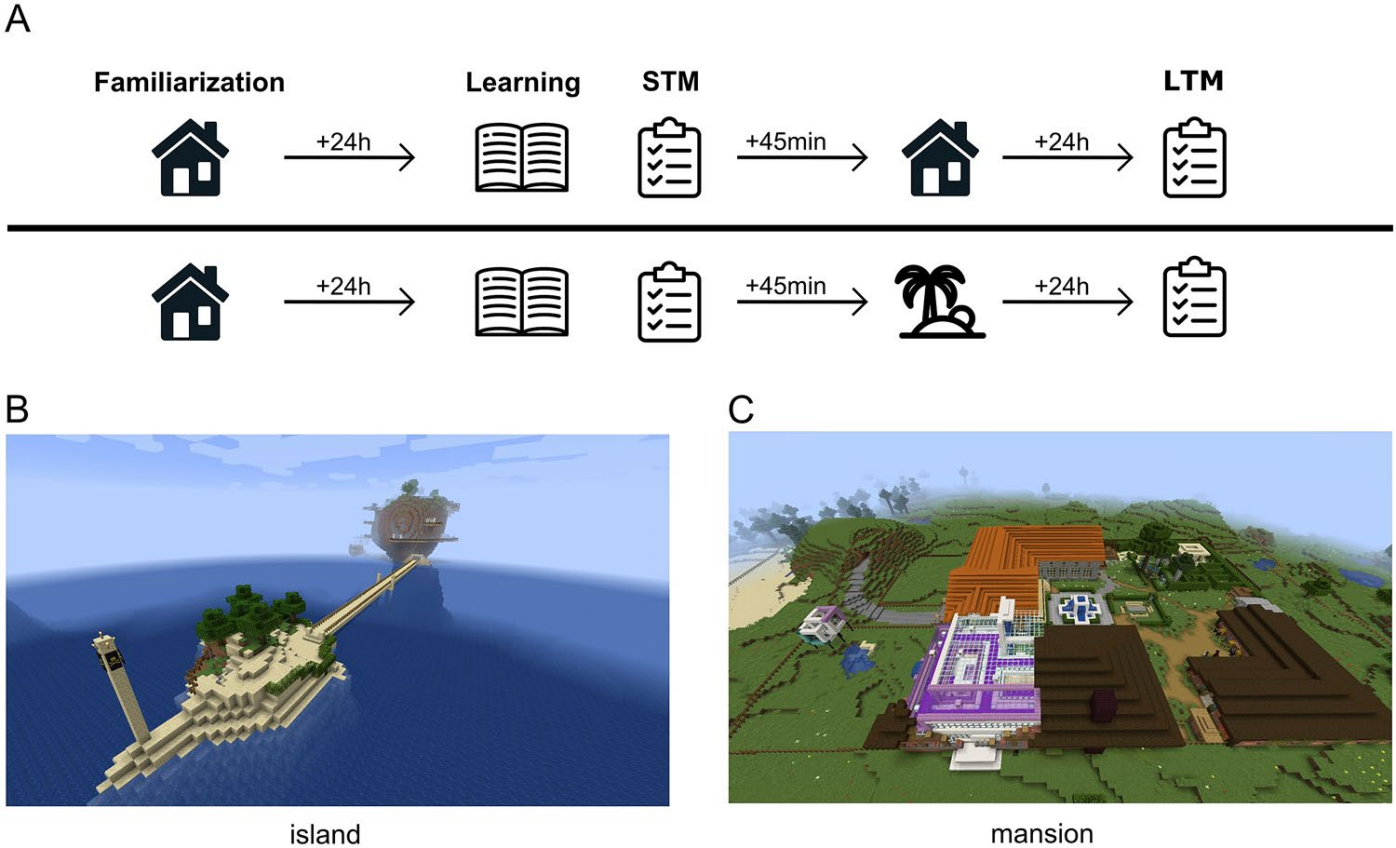
Overview over the experimental design (**A**) and the virtual environments presented in the experiment (**B** and **C**). On day 1, participants were familiarized with one of the two environments (familiarization). On day 2, participants at first learned a list of 20 words (learning) and had to immediately recall the words (STM). 45 min later, they explored either the already familiar environment (upper row) or a novel environment (lower row). On day 3, free recall was tested again (LTM). Note that in the design example above the “mansion” environment was familiarized, while we counterbalanced the order in which environments where presented in the experiment. The Minecraft save game data to recreate the environments is available at https://github.com/valentinbaumann/minecraft_adhd. STM = short term memory, LTM = long term memory.

Participants visited the lab on three consecutive days (Fig. 1A). To reduce novelty other than provided by the virtual environment every participant was tested in the same room and by the same experimenter on all three days. On day 1, children were familiarized with one of two virtual environments. We created the virtual worlds using the game “Minecraft” (version 1.12.2), with one world displaying a pirate-themed island (“island”, Fig. 1C) and the other a grand mansion including the surrounding grounds (“mansion”, Fig. 1B). Both environments were tested in pilot experiments to make sure they offered comparable experiences and environments rich enough to provide 10–20 min of exploration time (comparable to Ballarini et al.^29^), dependent on both how thoroughly participants would explore the world and how accustomed they were to controlling movement in the game. The environment was perceived through first person view and could be navigated freely using the “WASD” keys (default setting). All other keys were deactivated, as was all music except for environmental sounds like footsteps and animal noises. To keep lighting and atmosphere similar across all participants and sessions, the in-game time was always set back to 6 a.m. for every session. The “Raspberry Jam Mod” (https://github.com/arpruss/raspberryjammod/releases, Version 0.94) was used to log the player position every 100 ms.

Participants were shown how to navigate the avatar and instructed to explore the world, to thoroughly look around and to report back to the experimenter if they thought they had explored everything. Participants additionally were informed that they could not build or destroy anything and that there were no enemies they had to fight, which are common gameplay options in Minecraft. They were then allowed to freely explore the environment on their own. Since we expected major differences in how fast the participants would get used to controlling the avatar’s movements we did not determine a fixed amount of exploration time, but aimed for a range of 10 to 20 min. Therefore, children were verbally motivated (if necessary) to explore for a minimum of 10 min and had to stop if they exceeded 20 min of playing time. Additionally, children reported how often they played video games (“never”, “less than once per week”, “more than once per week” or “daily”), if they generally knew about the game Minecraft (“yes”, “no”) and how often they played Minecraft themselves (“never”, “less than once per week”, “more than once per week” or “daily”).

On day 2, participants started with learning a list of 20 common disyllabic words taken from a German elementary school dictionary^53^. None of the words appeared in the VLMT memory test used in the diagnostic procedure, and none of the words was directly related to the items and structures presented in the virtual environments. During learning, the words were presented in a random sequence in the center of the screen for a duration of 5 s for each word, separated by a 1.5 s presentation of a fixation cross. After all words had been presented once, participants could take a short break and continue to a second round, where again the complete list was presented in the same order as in the first round. Participants were instructed to try to remember as many words as possible, that the order of the words did not matter and that they would be tested immediately after the learning session. Additionally, participants were requested to read every word out loud to make sure their attention was directed to the learning material. Directly after learning, children and adolescents were asked to orally report all words they remembered in whatever order they preferred (STM recall), with the examiner writing down all answers.

Participants were then led to another room and told they could now take a break for the following 45 min. This room was already familiar from the diagnostic procedures. During the break, children and adolescents were allowed to occupy themselves with available toys. The use of mobile phones was not permitted during this time. To make sure potential group differences were not due to different attitudes toward novel experiences, participants additionally answered a paper and pencil version of the “novelty seeking” scale of the German adaptation^54^ of the Junior Temperament and Character Inventory (JTCI)^55^ during the pause.

After the pause, participants were pseudo-randomly matched to either the familiarity or the novelty condition, dependent on the results of the STM recall. This procedure created four groups: *TD-familiar, TD-novel, ADHD-familiar* and *ADHD-novel*. While matching resulted in a non-random allocation of participants to groups we considered this procedure necessary regarding our small sample size to ensure a comparable baseline performance between the familiar and novel conditions. Adhering to the same procedure as on day 1, participants in the familiarity condition then explored the already familiar environment, while subjects in the novelty condition explored the other, unknown environment. As novel virtual environments have been found to induce higher feelings of immersion which in turn contributed to memory performance^31^, we afterwards assessed subjective feelings of immersion using questions based on the Igroup Presence Questionnaire (IPQ)^56^.

Day 3 began with a free recall test of the participants’ memory for the word list (LTM recall). Children and adolescents then completed a recognition memory test (for results, see supplementary information). All computer tasks including Minecraft exploration were presented on a laptop (model: Dell Inspiron 17 7779, screen size: 17.3″, refresh rate: 60 Hz), learning and recognition task were presented via Presentation (Neurobehavioral Systems, Version 20.1).

### Statistical analysis

#### Estimating the effect of novelty on memory

For our statistical analysis, we chose a Bayesian approach instead of frequentist methods. If an analysis is based on a small sample size like the present one, this approach can be more powerful compared to traditional methods, especially if a model incorporates informative priors^57,58^. As a measure of memory consolidation, we computed the retention score (*retention* = hits LTM recall/hits STM recall * 100) indicating the percentage of correctly remembered words retained from STM to LTM. To investigate whether there was an effect of novelty on memory consolidation, we built a Bayesian linear model which predicted *retention* through the factors *diagnosis* (ADHD vs. TD) and *novelty* (familiar vs. novel), plus the interaction of diagnosis and novelty condition (*diagnosis *×* novelty*). The model estimated the posterior distributions for the *intercept*, the effect of *novelty*, the effect of *diagnosis* and the effect of the *novelty x diagnosis* interaction. Since in linear models with dummy coded categorical predictors the model effects are expressed in relation to a reference group, we additionally computed the posterior distributions of the other three experimental groups by adding the posterior distributions of the intercept and the posterior distributions of the respective effects. Subtracting the resulting group-specific distributions from another yielded the posterior distributions of the differences between groups. We used a weakly informative student-t prior for the intercept (*df* = 3, μ = 50, σ = 30), representing our knowledge from pilot experiments that children and adolescents with ADHD will most likely remember about half of the words from the learning session. For the model error term sigma, we used a weakly informative half student-t prior (*df* = 3, μ = 0, σ = 10). For the coefficients of *novelty* and *diagnosis* as well as the *novelty x diagnosis* interaction we used flat uniform priors, which gave an equal prior probability to positive as well as negative effects, including an effect of zero.

To determine if differences between groups were substantially different from zero, we computed the 95% Highest Density Interval (HDI) and compared it to a Region of Practical Equivalence (ROPE). This method is recommended to determine the size and significance of an effect^59–62^. Similar to the “new statistics” approach in the frequentist framework^60,63,64^, it relies on drawing conclusions directly from the posterior distribution, thus giving information on both size and uncertainty of an effect. The 95% HDI can be seen analogous to a 95% confidence interval in frequentist statistics and represents the 95% most likely values of the posterior distribution. The ROPE on the other hand is an interval that specifies the range of the data where an effect can be considered as “practical equivalent to zero”^59–62^. While the HDI is directly estimated from the data, the ROPE needs to be determined manually, as it depends on what the smallest effect size of interest is for the individual hypothesis. The default recommendation to determine the ROPE interval width is to set its upper and lower limits to ± 10% of the standard deviation of the underlying distribution, thus considering all values with an effect size smaller than 0.1 as equal to zero^65^. However, since *retention* was dependent on counts, in our data only group differences in *retention* that were greater than 5% represented a memory difference of more than one word, which we thought should be the minimal effect of interest. We therefore decided to set the ROPE limits to the more meaningful range of [− 5, 5]. The significance of an effect is represented by the overlap of ROPE and HDI. The more of the HDI lies outside of the ROPE, the greater is the evidence for the alternative hypothesis. According to Kruschke^65^, an effect can be considered significant if there is a probability of at least 95% that the effect is not practically equivalent to zero. This is the case when at least 95% of the posterior distribution (or all of the HDI) fall completely outside the ROPE. On the other hand, all parameter values covered by the ROPE can be considered to be practically equivalent to zero, since they fall below the minimal effect size of interest. Therefore, the more values lie inside the ROPE, the stronger the evidence is in favor of the null hypothesis. According to Kruschke^65^, the null hypothesis can be accepted if the ROPE covers at least 95% of the posterior distribution (equal to all of a 95% HDI), which indicates that the effect is practically equivalent to zero with a probability of at least 95%.

#### Analysis of control variables

To control for potential confounding variables, we analyzed whether participants in the four groups differed in their attitudes towards novel experiences, their feeling of immersion and their exploration behavior using two-way ANOVAs with the factors *diagnosis* (ADHD vs. TD) and *novelty* (novel vs. familiar). While participants filled in all items of the JTCI’s novelty seeking scale, we followed a previous approach by Fenker et al.^66^ and analyzed only the *exploratory excitability* subscale, as this subscale specifically measures the willingness to explore novel places and situations. Exploration behavior was quantified by collecting the position of each subject’s avatar every 100 ms. As an indicator for how much space a person explored, we divided both environments into tiles with a size of 2 × 2 Minecraft blocks and calculated how many unique tiles each person visited. Two participants were excluded from the exploration data analysis due to missing position datasets, while the data of two additional participants had to be rejected since they failed to correctly fill in the novelty seeking questionnaire. All statistical tests were conducted with an alpha level of α = 0.05.

#### Estimating the effect of exploration and immersion on memory

Since exploration, immersion and novelty seeking differed significantly between groups (“Influence of control variables” section), we additionally investigated whether these variables also influenced memory consolidation. As any potential influence could potentially depend on diagnosis as well as on whether a novel or familiar environment was explored, we created three Bayesian linear models to estimate how each of the control variables affected the individual groups. For *exploration*, we predicted memory retention by the number of unique tiles visited in interaction with diagnosis and novelty conditions (*retention* ~ *exploration* x *diagnosis* x *novelty*). To estimate the group specific effects of immersion and novelty seeking on memory retention we applied the same model but with immersion ratings (*retention* ~ *immersion* × *diagnosis* × *novelty*) or exploratory excitability ratings (*retention* ~ *novelty seeking* × *diagnosis* × *novelty*), respectively. Additionally, we z-transformed all three control variables (the number of unique tiles visited, immersion ratings and exploratory excitability ratings) to allow for a more meaningful interpretation of model effects.

All three models used the same priors for intercept and sigma as the main model (“Estimating the effect of novelty on memory” section). We assigned a flat uniform prior to all other model coefficients (*diagnosis* and *novelty*, plus *exploration*, *immersion* or *novelty seeking*, as well as any interaction terms), which gave an equal prior probability to positive as well as negative effects, including an effect of zero. To determine if there were any significant effects, we followed the same ROPE + HDI procedure as detailed in “Estimating the effect of novelty on memory” section. The ROPE limits were again set to [− 5, 5], which represents a minimal effect of five percent in retention per one standard deviation of *exploration, immersion* or *novelty seeking*.

For all Bayesian models, posterior distributions were sampled with four independent chains and 2000 samples per chain. The first 1000 samples of each chain were discarded as warm-up iterations. All chains converged for all three models ($\widehat{R}$ = 1 for all chains). Data analysis was conducted in *R* version 3.6^67^, using the packages *brms*^68^ and *bayestestR*^69^. Since the output generated by Bayesian models is based on random sampling processes, absolute values may vary slightly between repeated analysis runs. For a replication of the exact values a workspace image containing the samples on which the present analysis was conducted on can be found at https://github.com/valentinbaumann/minecraft_adhd. All plots were created in *R*^67^, using the package *ggplot2*^70^.

## Results

### Effect of novelty on memory

**Figure 2:**
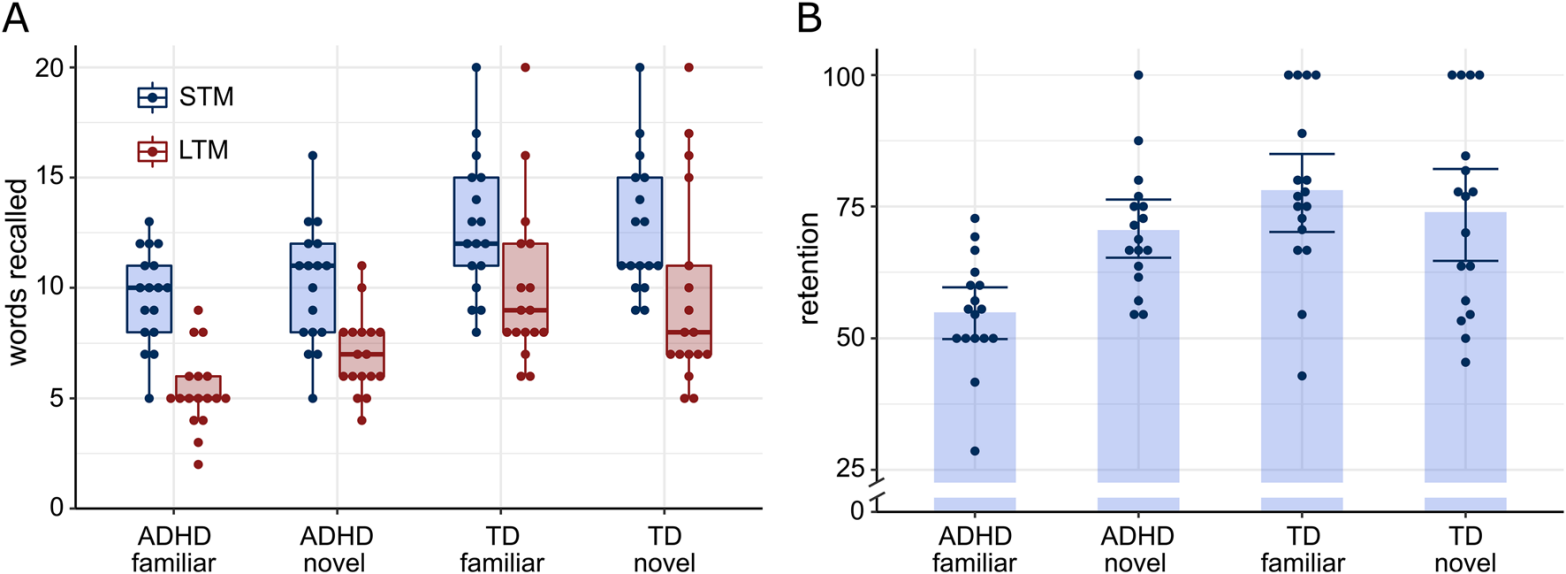
STM and LTM free recall scores with boxplots (**A**) and retention scores with means and 95% confidence intervals (**B**). Retention represents the percentage of words retained from STM to LTM (retention = LTM/STM * 100). Across a delay of 24 h, children and adolescents with ADHD retained a higher percentage of words if they explored a novel compared to a familiar environment (indicating improved memory consolidation). This effect was not observed for typically developing children and adolescents. Dots represent single participants, STM = short term memory, LTM = long term memory, TD = typically developing.

**Figure 3:**
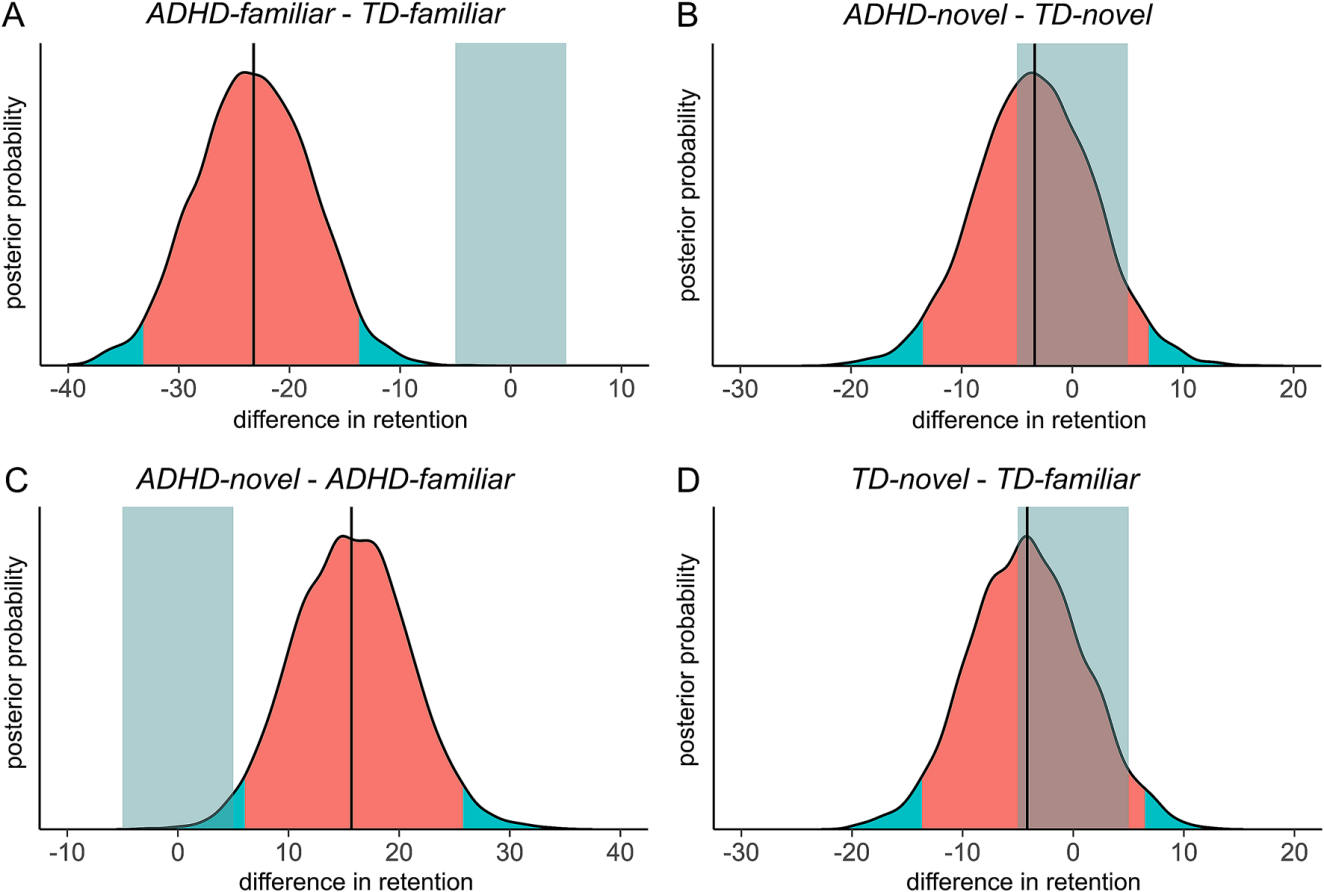
Posterior distributions of the differences in retention score between groups. The red area shows the 95% highest density interval (HDI), the light blue stripe represents the region of practical interest (ROPE) of [− 5, 5] and the black line indicates the mean of the posterior distribution. An effect can be considered to be significantly different from zero if more than 95% of the posterior distribution lie outside the HDI. Patients with ADHD initially showed a significant disadvantage in memory consolidation compared to typically developing children (**A**), but exploration of a novel environment alleviated this disadvantage (**B**). Direct comparison of the two patient groups indicated that patients who explored a novel environment retained a significantly higher percentage of words than patients who explored a familiar environment (**C**). We could not observe a significant memory benefit of novel environment exploration for typically developing children and adolescents. (**D**). Retention represents the percentage of words retained from STM to LTM (retention = LTM/STM * 100). TD = typically developing, STM = short-term memory, LTM = long-term memory.

To estimate the effect of novelty on memory, we ran a Bayesian linear model predicting consolidation performance as the percentage of words retained from STM to LTM by novelty condition and diagnosis (*retention* ~ *novelty* x *diagnosis*). Raw STM and LTM recall as well as retention scores are presented in Fig. 2. Patients who explored a familiar environment (*ADHD-familiar*) remembered on average *b* = -23.23% less words from STM to LTM recall than typically developing participants who explored a familiar environment (*TD-familiar*), 95% HDI [− 33.18, − 13.70]. Since 99.97% of the posterior distribution, including all of the HDI, lay outside the ROPE of [− 5, 5], memory retention for patients with ADHD was significantly impaired with a probability greater than 95% (Fig. 3A). Patients who explored a novel environment (*ADHD-novel*) remembered on average *b* = -3.39% less words from STM to LTM recall than typically developing participants who explored a novel environment (*TD-novel*), 95% HDI [− 13.53, 6.94]. Since only 43.57% of the posterior distribution lay outside the ROPE of [− 5, 5] we could not conclude that there was a significant difference between groups (Fig. 3B). Patients with ADHD who explored a novel environment (*ADHD-novel*) remembered on average *b* = 15.67% more words from day 1 to day 2 than patients who explored a familiar environment (*ADHD-familiar*), 95% HDI [6.03, 25.77]. 98.27% of the posterior distribution, including all of the values in the HDI, lay outside the ROPE of [− 5, 5]. This indicated that, with a probability greater than 95%, memory retention was significantly better if patients explored a novel environment (Fig. 3C). Typically developing children and adolescents who explored a novel environment (*TD-novel*) remembered on average *b* = -4.16% less words from day 1 to day 2 than typically developing participants who explored a familiar environment (*TD-familiar*), 95% HDI [− 13.69, 6.49]. Since only 48.70% of the posterior distribution lay outside the ROPE of [− 5, 5] our data suggested that exploration of a novel environment did not significantly improve memory for typically developing children and adolescents (Fig. 3D).

### Influence of control variables

**Figure 4:**
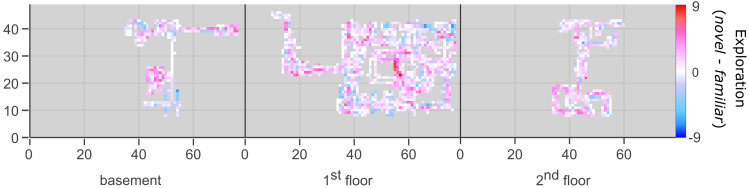
On day 2, participants explored more space in novel than in familiar environments (collapsed across *diagnosis*). The scale represents the difference in number of participants who visited a tile, with red tiles visited more often in the novel condition and blue tiles in the familiar condition. The first floor also included the starting area (left hand side) as well as a garden and stables (right hand side). Note that since the visualization only shows the “mansion” environment, it is based on only one half of the sample.

We additionally investigated if the individual attitude towards novelty exploration as well as the feeling of immersion into the virtual experience and the movement during virtual environment exploration differed between diagnosis and novelty groups. Children and adolescents with ADHD reported significantly higher levels of *exploratory excitability* than typically developing participants (main effect of *diagnosis), F*(1) = 6.42, *p* = 0.014, η_p_^2^ = 0.09. There was neither a significant main effect of *novelty* nor a significant interaction effect (all *p* > 0.38), indicating that the individual attitude towards novelty exploration did not differ significantly dependent on the novelty condition. For immersion, patients with ADHD reported significantly higher immersion ratings than typically developing children and adolescents (main effect of *diagnosis*), *F*(1) = 5.53, *p* = 0.022, η_p_^2^ = 0.08, but we did not observe a significant main effect of *novelty* nor a significant interaction effect (all *p* > 0.12), indicating that immersion ratings did not differ significantly dependent on whether a novel or a familiar environment was explored. Regarding exploration behavior on day 2, participants visited significantly more tiles if they explored a novel environment (main effect of *novelty*), *F*(1) = 7.68, *p* = 0.007, η_p_^2^ = 0.11 (Fig. 4). We observed neither a significant effect of *diagnosis* nor a significant interaction effect (all *p* > 0.38), indicating that exploration did not differ significantly between children and adolescents with and without ADHD. For the analysis of immersion ratings and exploration behavior, we collapsed values from both environment types (“mansion” and “island”). See Table 2 for the separate values of each environment type.

**Table 2:** Exploration and immersion on day 2 by environment types.

	Exploration (mansion)	Exploration (island)	Immersion (mansion)	Immersion (island)
ADHD-familiar	851.50 (177.97)	642.33 (126.50)	4.13 (4.97)	7.22 (12.09)
ADHD-novel	913.14 (188.90)	809.67 (144.15)	3.25 (14.16)	− 1.89 (9.48)
TD-familiar	875.63 ((169.20)	690.63 (141.90)	− 5.88 (10.29)	− 5.78 (12.12)
TD-novel	988.67 (139.17)	805.62 (108.20)	1.89 (16.48)	− 5.88 (14.67)

Exploration (number of tiles entered) and immersion ratings on day 2 for the four experimental groups and the two environment types. Values show means and standard deviations (in brackets). Immersion ratings can range from − 30 (min) to 30 (max). A detailed analysis of differences between environment types is given in the Supplementary Information.

**Figure 5:**
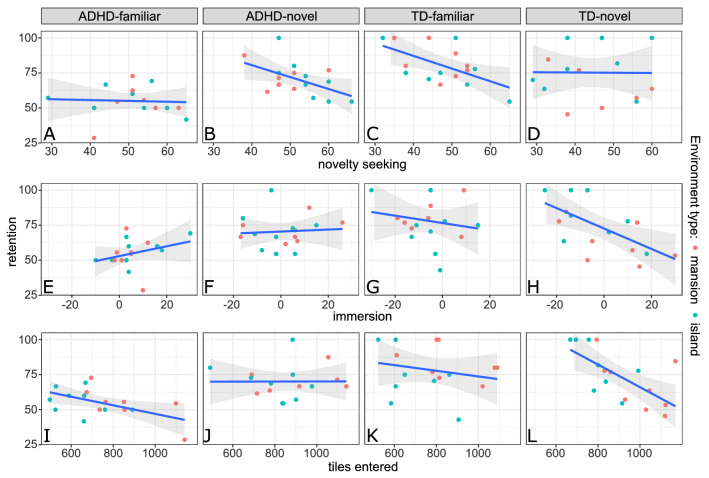
(**A**–**D**) Influence of *novelty seeking* on memory retention (exploratory excitability ratings, T-values), (**E**–**H**) influence of *immersion* (immersion ratings, raw scores) and (**I**–**L**) influence of *exploration* (number of tiles entered). In the TD-novel group, higher levels of exploration and immersion were associated with worse memory retention (**D**, **H**). Additionally, our data strongly suggested that this association was not present in the ADHD-novel group (**F**, **J**), indicating that the influence of exploration and immersion on memory retention might be different between children with and without ADHD. Note that here *exploration, immersion* and *novelty seeking* are shown on their original scales, while we z-transformed all three variables for the analyses reported in the text. Grey shaded areas indicate 95% confidence intervals, dots represent single participants. TD = typically developing, retention = LTM/STM * 100.

Since we observed significant group differences in our control variables, we additionally investigated if these variables affected memory consolidation in the different groups (Fig. 5, Table 3, Supplementary Fig. S1–S4). *Novelty seeking* did not significantly influence memory retention in any of the groups, since all HDIs showed considerable overlap with the ROPE (Fig. 5A–D). There were also no significant effects of *immersion* on memory retention in the *ADHD-familiar*, A*DHD-novel* and *TD-familia*r group (Fig. 5E–H). In contrast, typically developing children who explored a novel environment (*TD-novel*) remembered on average *b* = -9.15% less words for each standard deviation of *immersion,* 95% HDI [− 14.53, − 3.92] (Fig. 5H). While this effect was not significant according to the recommendations by Kruschke^65^, our model still suggested a very high probability of 93.55% for a negative influence of immersion on memory retention. For *exploration*, no significant association with memory retention could be observed in the *ADHD-familiar*, the *ADHD-novel* and the *TD-familiar* group (Fig. 5I–K). However, for each standard deviation of *exploration*, typically developing children who explored a novel environment (*TD-novel*) remembered on average *b* = -14.35% less words, 95% HDI [− 22.09, − 6.43] (Fig. 5D). There was a probability of 98.92% that this effect was significantly different from zero, since 98.92% of the posterior distribution including all of the HDI lay outside the ROPE of [− 5, 5]. Additionally, our model estimated a probability of 95.47% that this effect was significantly different from the effect of exploration in the *ADHD-novel* group, *b*_Δ_ = -14.40, 95% HDI [− 25.04, − 3.10]. This indicated that, for novel environments, the relationship of exploration and memory retention was different between children and adolescents with and without ADHD.

**Table 3:** Control variables and their influence on memory consolidation.

Variable	Mean (SD)	Slope (*b*)	95% HDI	% of posterior distribution outside ROPE of [− 5, 5]
**Novelty seeking**^**a**^				
ADHD-familiar	54.82 (12.33)	− 0.45	[− 7.29, 6.23]	14.08
ADHD-novel	56.82 (7.92)	− 7.80	[− 15.96, 0.98	74.37
TD-familiar	43.00 (6.78)	− 8.19	[− 15.17, − 0.83]	86.65
TD-novel	40.25 (10.08)	− 0.06	[− 6.20, 5.96]	9.07
**Immersion**^**b**^				
ADHD-familiar	5.76 (9.30)	4.34	[− 4.31, 13.88]	47.07
ADHD-novel	0.53 (11.82)	0.88	[− 6.73, 7.93]	18.70
TD-familiar	− 5.82 ( 10.94)	− 3.35	[− 11.03, 4.63]	36.42
TD-novel	− 1.76 (15.68)	− 9.21	[− 14.21, − 3.22]	93.57
**Exploration**^**c**^				
ADHD-familiar	740.76 (182.87)	− 5.37	[− 12.13, 0.95]	55.17
ADHD-novel	854.94 (167.83)	0.04	[− 7.73, 7.37]	17.40
TD-familiar	783.12 (178.56)	− 3.61	[− 10.56, 3.10]	35.30
TD-novel	902.53 (153.87)	− 14.35	[− 22.09, − 6.43]	98.92

Means and standard deviations (in brackets) for the three control variables, as well as slopes and HDI for the association of control variables and memory retention. The slope indicates the change in memory retention for one standard deviation of the respective control variable. The percentage of the posterior distribution that lies outside the ROPE represents the probability that an effect is significantly different from zero. Values above 95% indicate that an effect can be considered significantly different from zero, while values below 5% indicate that the effect can be considered practically equivalent to zero^65^.

*HDI* highest density interval, *ROPE* region of practical equivalence, *TD* typically developing.

^a^Exploratory excitability ratings (T-values).

^b^Immersion ratings (raw scores).

^c^Number of tiles entered.

## Discussion

### Novel environment exploration improves memory in ADHD

The present study aimed to investigate how the exploration of a novel environment impacts episodic memory consolidation in children and adolescents with and without ADHD. Our results show that in patients with ADHD intentional learning of a word list can be enhanced by the subsequent exploration of an unrelated novel virtual environment (Fig. 3C). Positive effects of novel experiences on memory are well known and in line with current functional models of novelty processing^71–74^. In our experiment memory could be enhanced even though the novel experience was presented 45 min after encoding. This strengthens the idea of a behavioral tagging and capture process, where during a sensitive period of consolidation salient stimuli are able to influence the synaptic plasticity processes underlying memory formation^14–16^. Until now, this behavioral tagging effect has been mostly investigated in animal experiments with novel environment exploration as the salient stimulus^19,22,75,76^. Our study shows that virtual novel environment exploration can be used to translate behavioral tagging to humans. Moreover, while behavioral tagging previously has been discussed to be a potential way to improve learning in typically developing school-aged children^29,30^, our data suggests that behavioral tagging can also be used to overcome memory problems in children and adolescents with ADHD (Fig. 3A,B).

Interestingly, contrary to our expectations, typically developing participants did not significantly profit from novel environment exploration (Fig. 3B). This contradicts observations made in previous experiments, which found a positive effect of novelty on memory in both healthy adults^31,32^ as well as typically developing children and adolescents^29,30^. One possible explanation is that our word list task produced a learning event in typically developing participants that was already too strong for the novel experience to be effective. According to behavioral tagging, the exposure to a novel environment provides additional PRPs that can be captured by weakly stimulated synapses. A strong learning event however already generates a sufficient amount of PRPs and cannot be improved further by behavioral tagging. Behaviorally, weaker learning should be evident in decreased memory retention. In the familiar condition, our analysis of retention performance indeed indicated weaker encoding for patients with ADHD compared to typically developing children and adolescents (Fig. 3A).

Neurobiologically, this could be due to an hypofunction of the dopaminergic system in ADHD, which is specifically expressed in low tonic dopamine (DA) levels^77–81^. The dopaminergic system is crucial for memory formation, as hippocampal DA presumably modulates PRP synthesis and has shown to be necessary to establish late LTP^75,82–84^. While in ADHD there is no evidence of altered dopaminergic neurotransmission directly in the hippocampus, several studies indicate that the dopaminergic structures projecting to the hippocampus are affected, especially the substantia nigra (SN) and the ventral tegmental area (VTA)^85–89^. Together with the locus coeruleus (LC), these structures control the release of DA in the hippocampus and thus mediate the encoding of salient information^74,82,83,90^. Importantly, functional models of novelty processing predict that experiencing novelty, for example the exploration of a novel environment, leads to an increase in the number of tonically firing neurons in the SN and VTA^71,72,91,92^. While in ADHD initial low tonic DA levels might have led to weak encoding, the exploration of a novel environment therefore could have increased DA levels in the VTA/SN, which in turn would upregulate DA release in the hippocampus, followed by heightened PRP synthesis and improved memory consolidation. However, in typically developing children, DA levels and encoding performance already could have been at an optimal level, so the additional release of DA caused by the novel environment might not have led to memory enhancement. We observed a similar ADHD specific improvement in response to salient stimuli in two previous studies^6,93^. In these experiments, children and adolescents with and without ADHD had to memorize both neutral and salient pictures. While in patients memory of neutral pictures was significantly impaired, they performed on an equal level to the control group if items were salient, indicating that the effect of a salient experience was proportionally greater if initial encoding capability was weaker. Interestingly, Ballarini et al.^29^ found that the positive effect of a novel school lesson on story learning only showed in hard-to-remember details, but not in easier, more general aspects of the story. This implies that the behavioral tagging effect might indeed be dependent on how deep a memory is already encoded.

Rodent experiments also show that behavioral tagging can depend upon initial encoding strength. Here, the strength of the learning event is often varied through the shock intensity used to train animals in an inhibitory avoidance task. In a study by Moncada and Viola^19^, the exploration of a novel open field improved memory retention when the shock intensity was low (0.15 mA), but left retention unaffected when the shock intensity was high (0.4 mA). Other experiments using shock intensities of 1.0 mA even observed that novelty exploration impaired memory retention^94,95^. A similar dependence has also been shown for other salient experiences used in behavioral tagging studies. For example, several animal studies and one human experiment reported that post-encoding stress improved weakly encoded memories, but impaired strongly encoded ones^96–99^.

We propose that the lack of effect in our TD-novel group could be due to the differing task requirements in our and previous studies. First, to keep ecological validity high, we used intentional word list learning while other human behavioral tagging studies like Ballarini et al.^29^ or Ramirez Butavand et al.^30^ employed incidental learning tasks without the explicit instruction to learn or the indication of a subsequent memory test. Intentional learning results in stronger encoding than incidental learning, mostly because it leads to the application of mnemonic strategies, for example clustering items into categories or creating associative stories^100,101^. On the other hand, intention to learn can be irrelevant if the use of mnemonic strategies is blocked, for example by providing less time per item or adding an additional orienting task^100,102^. The latter example might explain why, in contrast to our experiment, a study by Fenker et al.^32^ found an enhancing effect of novel images on intentional learning. In this experiment subjects were explicitly instructed to learn a list of words but additionally had to categorize the words as either living or nonliving. The resulting encoding strength might therefore be more similar to that of the incidental learning task used by Ballarini et al.^29^ than to the task used in the present study. Taken together, typically developing children and adolescents were probably able to profit from the intentional nature of the learning task. Patients with ADHD on the other hand are known to make less use of mnemonic strategies and in general show difficulties in allocating and maintaining effort to a task^103,104^. This is in line with our observation that the majority of the control group reported the use of strategies, but only very few patients.

The second methodological difference that might have led to strong instead of weak encoding might be that we presented an immediate free recall test directly after encoding of the word list. A similar measure of STM memory was present neither in the study by Ballarini et al.^29^ nor the studies by Ramirez Butavand et al.^30^ or Fenker et al.^32^. Retrieval of an encoded stimulus can improve memory of that stimulus and this “testing effect” has been shown to be even greater than the effect of restudying^105,106^. Since we tested both STM and LTM in the same sample, individuals from our control group were probably able to profit from this retrieval effect and thus strengthened their memory of the word list. On the other hand, patients with ADHD again might have benefitted less from the additional memory test than typically developing participants, as a study with young adults with ADHD indicates that patients profit less from the testing effect than a healthy control group^107^.

The experiment by Schomaker et al.^31^ also differs in some crucial methodological aspects. Similar to our study, subjects explored either a familiar or a novel virtual environment and the learning task consisted of intentional learning of a word list. However, in contrast to our study, exploration occurred directly before learning, which allows the novel environment to influence both consolidation as well as initial encoding. The experiment also only included only an immediate, but not a delayed recall. Since behavioral tagging relies on modulation of memory consolidation, differences in memory due to behavioral tagging should only be evident after a delay. It is therefore unclear whether the effect observed by Schomaker et al.^31^ is also attributable to behavioral tagging or solely to a modulation of initial learning, for example by an increase in motivation or arousal^31,108,109^.

### The individual experience of a novel environment influences memory in typically developing children

To control for potential differences between diagnosis and novelty conditions, we additionally assessed the individual attitude towards novel experiences as well as the feeling of immersion into the virtual experience and the movement behavior during environment exploration. Patients with ADHD showed a significantly higher readiness to explore novel situations and places, which is a common observation in the literature^110–112^. This personality trait did not significantly predict memory retention in any of the experimental groups (Fig. 5A–D), indicating that the individual attitude towards novel experiences did not mediate the behavioral tagging effect. This is in line with the observation made in a very recent study where novelty seeking also did not influence how the exploration of a novel or familiar environment altered memory performance^37^.

Children and adolescents with ADHD also reported significantly higher immersion. The level of immersion was previously associated with how many attentional resources are devoted to the virtual experience^113^, as well as motivational involvement^31^. While in a previous study higher immersion ratings were related with better memory performance^31^, we observed no significant effect of immersion on memory retention in any of the groups with the exception of typically children who explored a novel environment. Here, our data suggested that higher levels of immersion negatively influenced memory retention (Fig. 5H). This difference in effects might be caused by two factors. First, the environments in the experiment by Schomaker et al.^31^ were presented via virtual reality headsets, which could induce a different quality of immersion. Second, the virtual environments were presented closely before learning, while we presented our environments 45 min after learning. Whether immersion causes positive or negative effects might therefore also depend on the timeframe in which the virtual experience is presented.

For movement behavior, both children and adolescents with and without ADHD explored significantly more space if they played a novel compared to a familiar environment. We again found no effect on memory retention in any of the groups with the exception of typically children who explored a novel environment. Here, we also observed significantly worse memory retention the more tiles participants discovered (Fig. 5L).

Although we had no a priori hypothesis for these observations, we propose it could be caused by memory traces of the environment and the word list interfering with each other. Human studies on memory interference show that interference effects are larger the more competing items are presented and the better these items are encoded^114–118^. Participants who discovered more parts of an environment potentially could have created a richer memory trace for the environment, leading to stronger interference. Similarly, participants who were comparatively more immersed into playing Minecraft also potentially acquired a more complex memory trace of the environment, resulting in increased interference.

Interestingly, for both immersion and exploration, we found this effect only in typically developing participants exploring a novel environment. It might be possible that creating a new memory trace for a novel environment in general is more interfering than the mere reactivation of an old memory, as previous animal studies observed that only novel, but not familiar environments interfered with memory of a preceding task^95^. However, while this might explain why we did not observe significant effects for children and adolescents exploring familiar environments, it does not account for the apparent lack of interference in the *ADHD-novel* group. *ADHD-novel* was also the only group where the influence of exploration on memory retention was significantly different from the effect in the *TD-novel* group (Fig. 5J,L), with immersion showing a similar pattern (Fig. 5F,H). As the absolute values of both exploration and immersion were equal or higher in the patient groups (Table 3), it is possible that the interference effect itself differed between children and adolescents with and without ADHD. This might be due to atypical consolidation processes in ADHD, as a recent study observed that an interference task presented 2 h after training in a procedural memory tasks only impaired memory consolidation for typically developing children^119^. However, to our knowledge, there are no similar studies that would indicate that episodic memory consolidation in children with ADHD also could be less susceptible to interference. An alternative explanation might therefore be that, while we did not issue any instruction to learn anything about the environments, some typically developing subjects nevertheless expected the environments to be part of a future memory test and therefore tried to memorize their content. While the memory trace of the environment in general could have become more complex the more participants explored and allocated attentional resources to the virtual world, the intention to deliberately learn environment details might have a led to a stronger level of encoding for typically developing participants that ultimately caused the observed interference effect. To obtain a better understanding of such an interaction, it could be interesting to measure not only memory of the initial learning task, but also memory of the novel environment in future experiments.

### Summary

In the present experiment, we showed that the exploration of a novel virtual environment improved word list learning in children and adolescents with ADHD. However, we did not find an improvement for typically developing children, implying that behavioral tagging might be most promising for individuals that naturally show weaker learning. Moreover, our data suggested that increased exploration of a novel environment as well as higher feelings of immersion compromised memory in typically developing children and adolescents, but left patients with ADHD unaffected. Getting a better understanding of what factors drive beneficial and detrimental effects of exploring a novel environment is therefore central to create future tailored memory intervention based on behavioral tagging.

## Supplementary information

Supplementary Information.

## Data Availability

The datasets generated during the current study are publicly available on GitHub, as well as all files necessary to recreate the analysis and the experiment itself [https://github.com/valentinbaumann/minecraft_adhd].
