# Supplementary material for: Exploration of a novel virtual environment improves memory consolidation in ADHD
Source: Sci Rep. 2020 Dec 8;10:21453. doi: 10.1038/s41598-020-78222-4 (PMC7722922; doi:10.1038/s41598-020-78222-4)
Supplement: Supplementary file 1 — Supplementary Information. [file 41598_2020_78222_MOESM1_ESM.docx]

**Exploration of a novel virtual environment improves memory consolidation in ADHD**

--- Supplementary file ---

Valentin Baumann^1*^, Thomas Birnbaum^1^, Carolin Breitling-Ziegler^1^, Jana Tegelbeckers^2^, Johannes Dambacher^1,3^, Elke Edelmann^4,6^, Jorge Bergado-Acosta^5,6^, Hans-Henning Flechtner^1^, Kerstin Krauel^1,6^

^1^Department of Child and Adolescent Psychiatry and Psychotherapy, University of Magdeburg, Germany

^2^Department of Neurology, Feinberg School of Medicine, Northwestern University, Chicago, IL, USA

^3^Faculty of Computer Science, University of Magdeburg, Germany

^4^Department of Physiology, University of Kiel, Germany

^5^Department of Pharmacology and Toxicology, University of Magdeburg, Germany

^6^Center for Behavioral Brain Sciences, Magdeburg, Germany

* Address: Department of Child and Adolescent Psychiatry and Psychotherapy, Leipziger Straße 44, 39120 Magdeburg, Germany. E-mail: valentin.baumann@med.ovgu.de

## Posterior distributions for the model *retention* ~ *exploration* x *diagnosis* x *novelty*


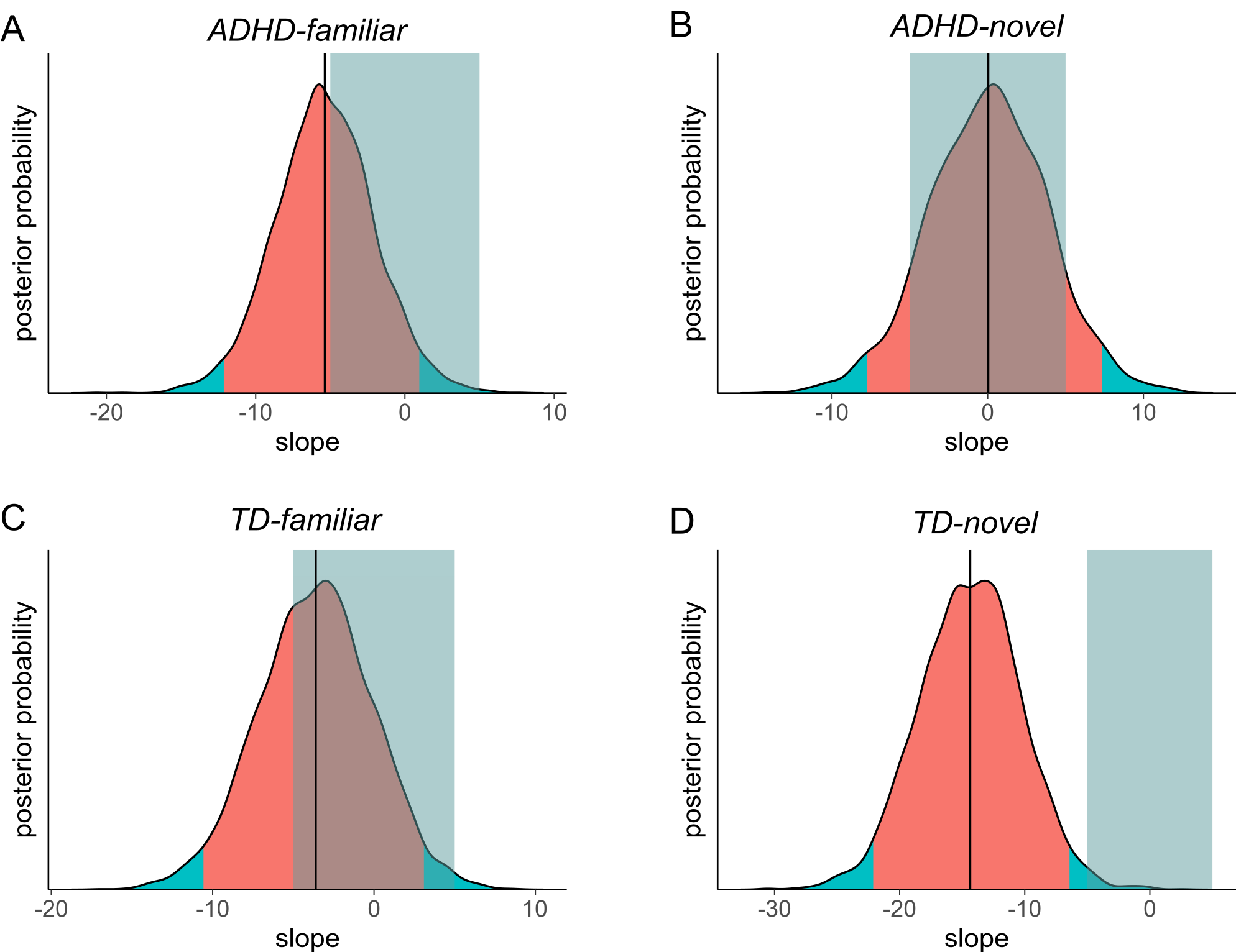


**Fig. S1.** Posterior distributions for the slopes of *exploration* for the ADHD-familiar (**A**), ADHD-novel (**B**), TD-familiar (**C**) and TD-novel group (**D**). The slope indicates the absolute change in *retention* (percentage of words remembered from STM to LTM) per standard deviation of *exploration* (number of unique tiles visited). The red area is the 95% HDI, the light blue stripe represents the ROPE and the black line indicates the mean of the posterior distribution. If more than 95% of the posterior distribution lie outside the HDI, the effect can be interpreted as significantly different from zero.


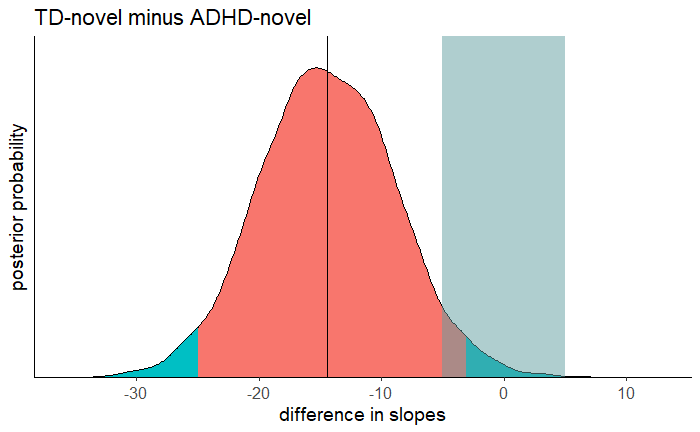


**Fig. S2.** Posterior distribution for the difference (*b*_Δ)_ between the slopes of *exploration* in the *TD-novel* and the *ADHD-novel* group (*b*_Δ_ *= b*_TD-novel_ - *b*_ADHD-novel_). The red area is the 95% HDI, the light blue stripe represents the ROPE of [-5, 5] and the black line indicates the mean of the posterior distribution. If more than 95% of the posterior distribution lie outside the HDI, the effect can be interpreted as significantly different from zero. If the ROPE covers the HDI completely, the effect can be considered as practically equivalent to zero.

## Posterior distributions for the model *retention* ~ *immersion* x *diagnosis* x *novelty*


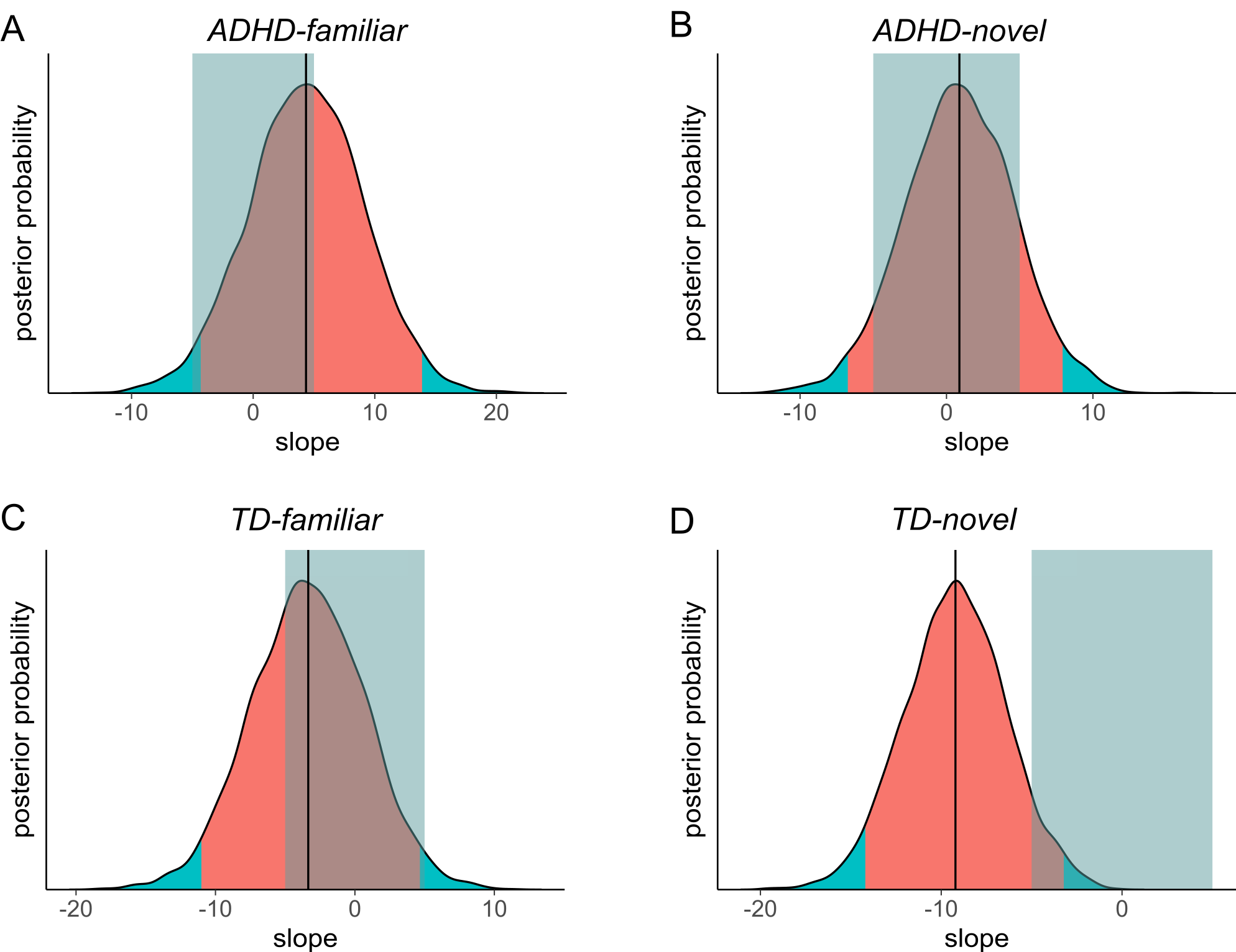


**Fig. S3.** Posterior distributions for the slopes of *immersion* for the ADHD-familiar (**A**), ADHD-novel (**B**), TD-familiar (**C**) and TD-novel group (**D**). The slope indicates the absolute change in *retention* (percentage of words remembered from STM to LTM) per standard deviation of *immersion*. The red area is the 95% HDI, the light blue stripe represents the ROPE and the black line indicates the mean of the posterior distribution. If more than 95% of the posterior distribution lie outside the HDI, the effect can be interpreted as significantly different from zero.

## Posterior distributions for the model *retention* ~ *novelty seeking* x *diagnosis* x *novelty*


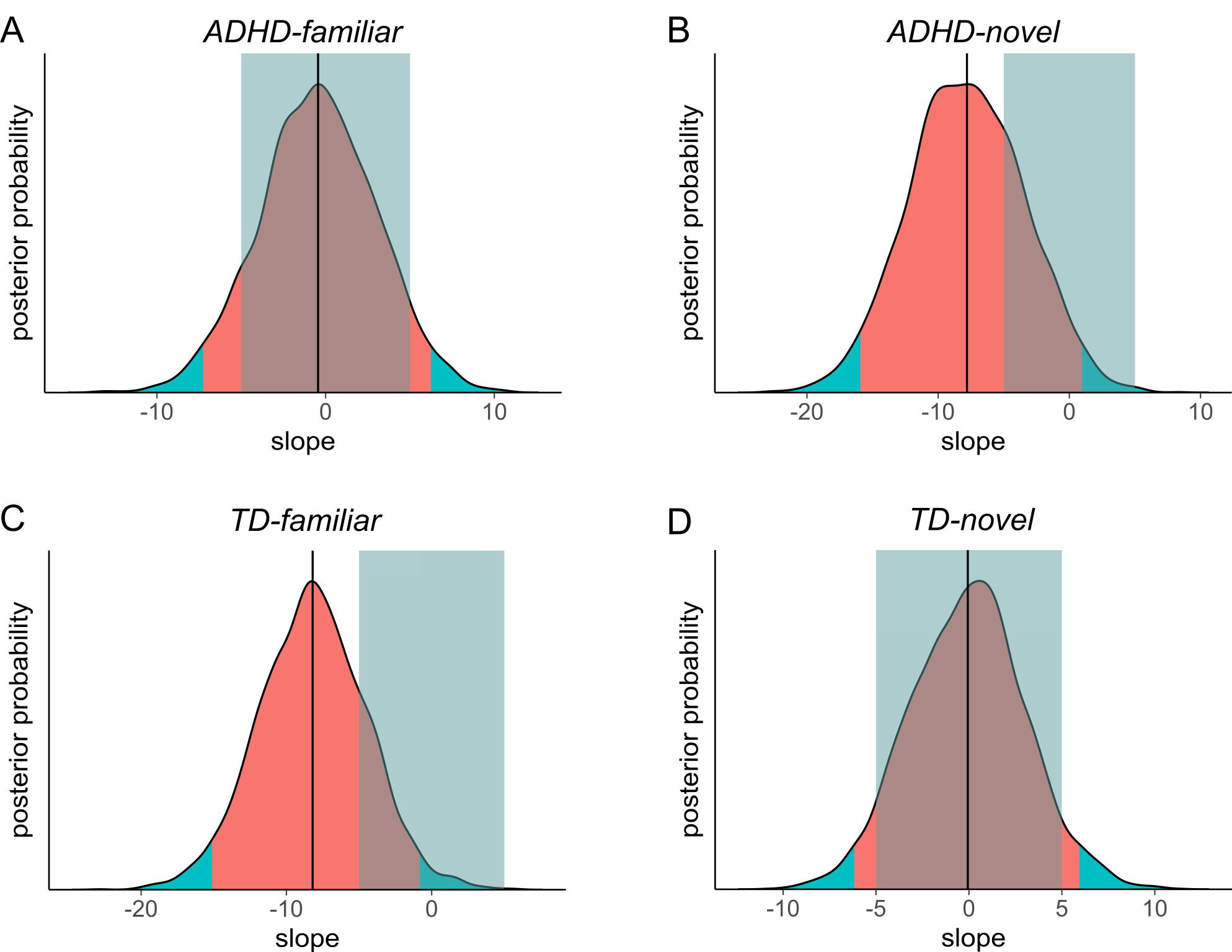


**Fig. S4.** Posterior distributions for the slopes of *novelty seeking* for the ADHD-familiar (**A**), ADHD-novel (**B**), TD-familiar (**C**) and TD-novel group (**D**). The slope indicates the absolute change in *retention* (percentage of words remembered from STM to LTM) per standard deviation of *novelty seeking*. The red area is the 95% HDI, the light blue stripe represents the ROPE and the black line indicates the mean of the posterior distribution. If more than 95% of the posterior distribution lie outside the HDI, the effect can be interpreted as significantly different from zero.

## Recognition data

### Methods

We presented all 20 words that children had learned on day 2 intermixed with 20 distractor words (random order). Distractor words were taken from the same source as the words in the learning task. Words were presented for 5 s each, separated by 1.5 s fixation trials. Participants were instructed to judge whether the present word was a part of the learning list on the day before or if it was a new word. Subjects were told that they should answer as fast and accurately as possible and that during presentation of the word they could correct themselves by pressing the desired button again. Participants indicated their decision via the left and right arrow keys on the keyboard. The allocation of keys to responses was determined randomly for every participant.

### Analysis

Recognition accuracy was computed as corrected hitrate (*hitrate* – *false alarm rate)*. To analyze if exploration of a novel or familiar environment influenced recognition accuracy, we built a model predicting accuracy by diagnosis group and novelty condition as well as their interaction (*accuracy* = *diagnosis* x *novelty*).

As we did not have prior data on recognition performance, we used default priors set by *brms* for the intercept (student-t, *df* = 3, μ = 1, σ = 10), as well as for the model error term sigma (half student-t, *df* = 3, μ = 0, σ = 10). For the coefficients of *novelty* and *diagnosis* as well as the *novelty x diagnosis* interaction we used flat uniform priors, which gave an equal prior probability to positive as well as negative effects, including an effect of zero. Posterior distributions were sampled using the NUTS algorithm with four independent chains and 2000 samples per chain. The first 1000 samples of each chain were discarded as warm-up iterations. All chains converged (for all chains, $\hat{R}$ = 1). We computed the overlap between HDI and ROPE to determine if differences between groups were significant (Kruschke, 2018). The ROPE was set to [-0.05, 0.05], representing a minimal difference of at least one more word recognized correctly or one less false alarm.

### Results

Figure S5 and table S1 display raw data and summary statistics. Our model reported that children and adolescents with ADHD on average recognized the words less accurately than typically developing children, *b* = -.21, 95% HDI [-.27, -.02]. This main effect of diagnosis was significant with a probability of 96.00 % (Fig. S6 E). Novel environment exploration had neither a significant effect on patients, *b* = .01, 95% HDI [-.11, .13], *P*(significant difference) = 41%, nor typically developing participants, *b* = .01, 95% HDI [-.10, .14], *P*(significant difference) = 42% (Fig. S6 C and D).

**Table S1: Recognition memory**

|  | ADHD-familiar | ADHD-novel | TD-familiar | TD-novel |
| --- | --- | --- | --- | --- |
| Hits | 15.82 (2.48) | 16.59 (3.02) | 17.82 (1.88) | 17.12 (2.5) |
| False alarms | 2.41 (2.40) | 2.94 (2.41) | 2.41 (2.18) | 1.41 (1.84) |
| Accuracy | .67 (.18) | .68 (.19) | .77 (.13) | .79 (.18) |

Scores depict means and standard deviations (in brackets). Accuracy = hitrate – false alarm rate.


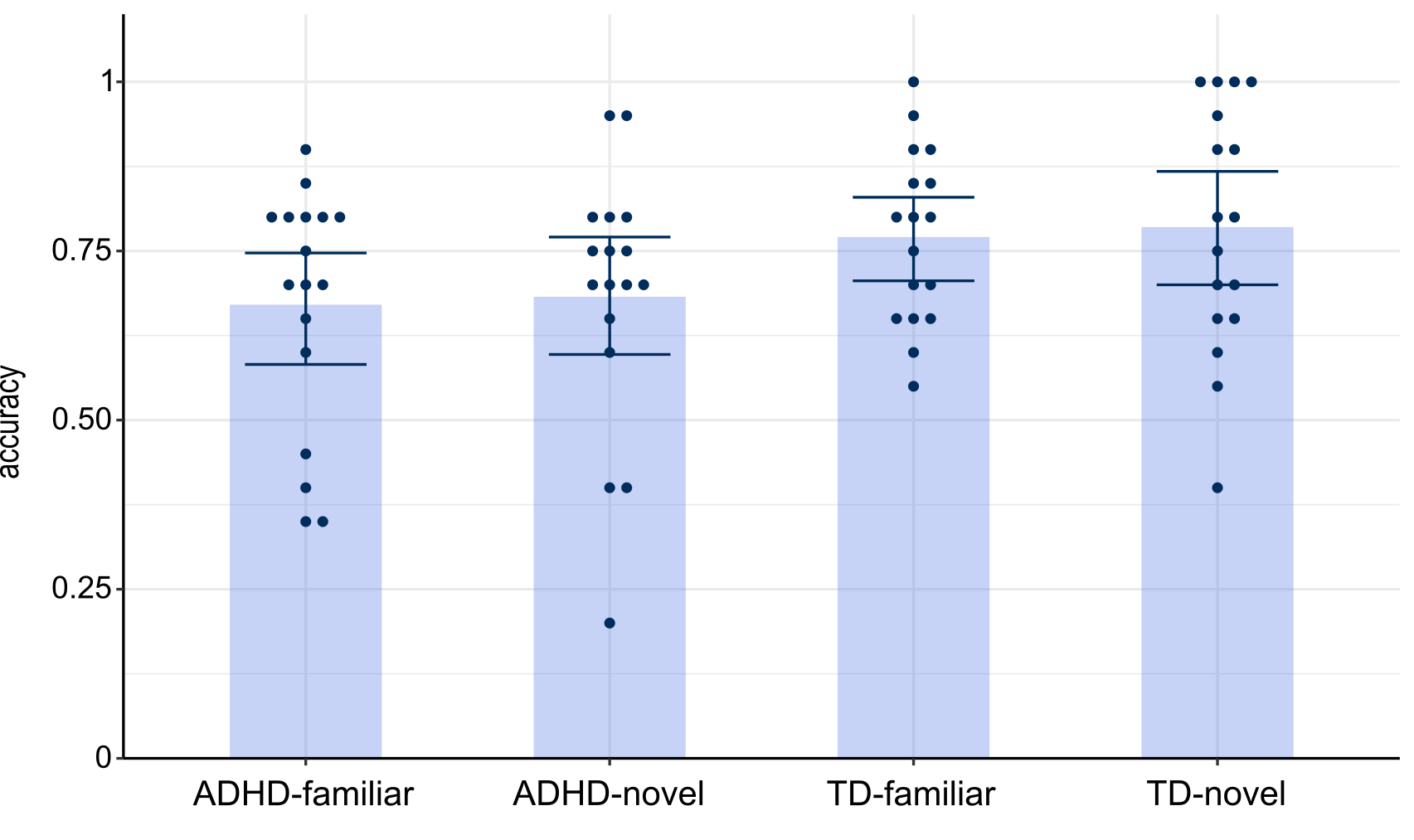


**Figure S5.** Accuracy (accuracy = hitrate – false alarm rate) for all groups (means and 95% confidence intervals). Typically developing subjects recognized words more accurately, while there was no effect of novelty. Dots represent single subjects, TD = typically developing.


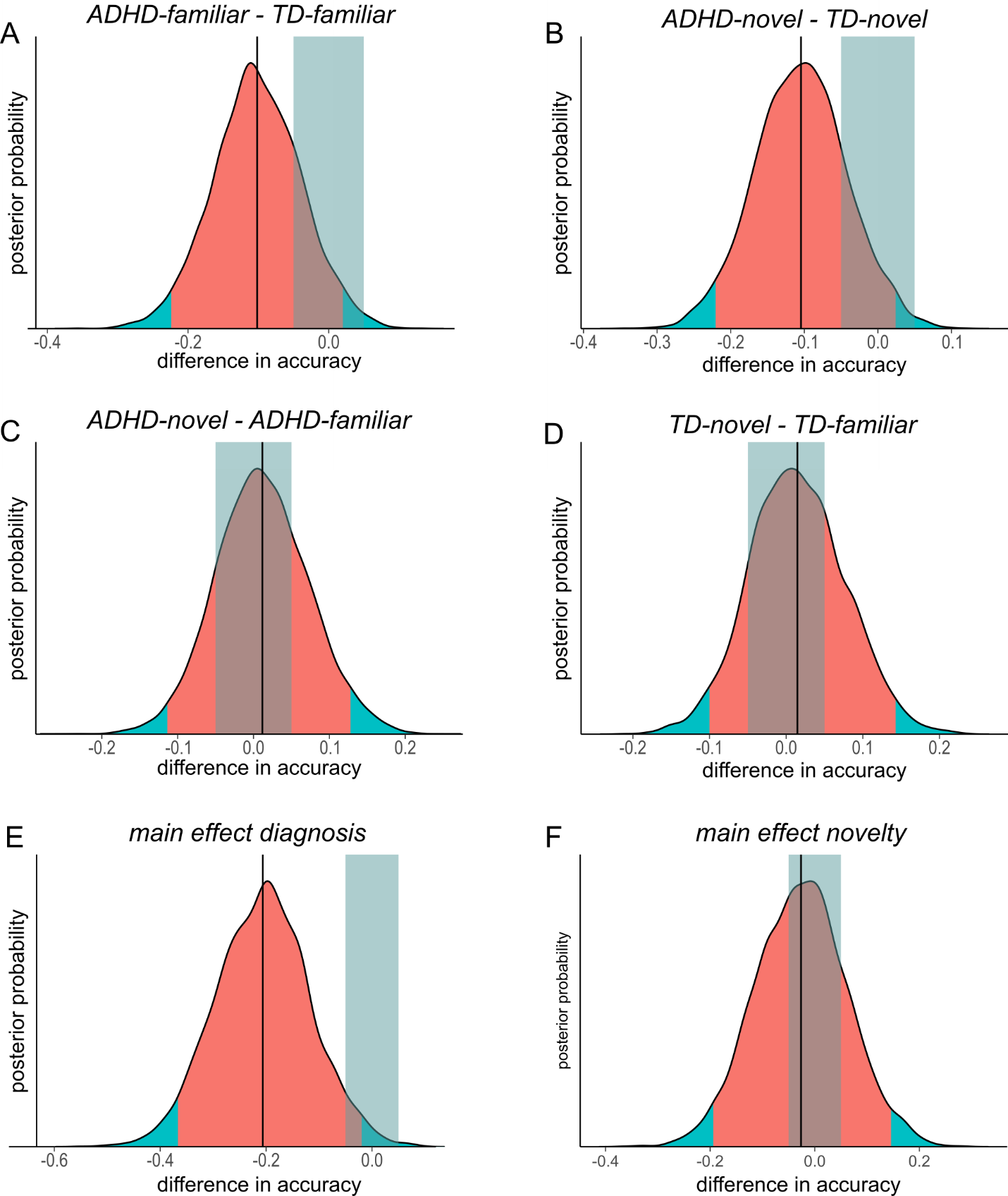


**Figure S6.** Posterior distributions of the differences in retention score between ADHD-familiar and TD-familiar (**A**), ADHD-novel and TD-novel (**B**), ADHD-novel and ADHD-familiar (**C**), TD-novel and TD-familiar (**D**) as well as the posterior distributions of the main effects of diagnosis (**E**) and novelty (**F**). The red area shows the 95% highest density interval (HDI), the light blue stripe represents the region of practical interest (ROPE) of [-5, 5] and the black line indicates the mean of the posterior distribution. An effect can be considered to be significant if more than 95% of the posterior distribution lie outside the HDI. We observed a significant main effect of diagnosis indicating that patients regardless of the novelty condition recognized the words less accurately than typically developing participants (**E**). There was neither a significant effect of novelty for children and adolescents with nor without ADHD (**F**, **C**, **D**). Main effect of diagnosis = ADHD-novel + ADHD-familiar - TD-novel - TD-familiar, main effect of novelty = ADHD-novel + TD-novel -TD-familiar - ADHD-familiar, TD = typically developing.

## Comparability of environment types (“mansion” vs. “island”)

Across all groups, there was no significant difference in exploration time between the two environments when measured at day 1, *t*(63.20) = 1.65, *p* = .10, 95% CI [-0.35, 3.73]. However, on day 1, participants explored significantly more space in the “mansion” environment, as indicated by a higher number of tiles discovered, *t*(63.58) = 4.22, *p* < .01, 95% CI [98.34, 274.97]. Despite the difference in exploration, there was no significant difference in retention score in regards to which environment was presented at day two, *t*(65.97) = -0.23, *p* = .82, 95% CI [-9.17, 7.25]. Across diagnosis and novelty conditions, there also was no significant difference in immersion ratings between the environment types on day 2, *t*(65.92) = 0.76, *p* = .45, 95% CI [-3.79, 8.46].

## Data and Scripts

Experiment files, raw data and analysis scripts to replicate experiment and analysis are available at <https://github.com/valentinbaumann/minecraft_adhd>.
